# Reaction strategies before and after the initiation of counterattacks in elite and sub-elite Kendo athletes

**DOI:** 10.3389/fspor.2026.1947221

**Published:** 2026-10-21

**Authors:** Kenshiro Matsuzaki, Takahiro Nabeyama, Yuji Arita, Seiji Ono, Tomohiro Kizuka

**Affiliations:** Institute of Health and Sport Sciences, University of Tsukuba, Ibaraki, Japan

**Keywords:** peak acceleration, peak jerk, premature reaction, pre-movement muscle activity, response accuracy, RT

## Abstract

**Objective:**

This study examined how differences in high-level expertise are reflected across multiple aspects of the reaction process during Kendo counterattacks.

**Methods:**

Eighteen male collegiate Kendo athletes (elite, *n* = 9; sub-elite, *n* = 9) performed counterattacks in response to opponent videos. Shinai motion was recorded with an acceleration sensor, and surface EMG was obtained from three upper-limb muscles. Reaction time (RT) was defined as the interval between the onset of opponent movement and the initiation of the counterattack. Premature reactions-transient acceleration fluctuations detected prior to counterattack initiation-were identified separately from the movement onset used to calculate RT. Peak acceleration and peak jerk after movement onset were also calculated, and pre-movement muscle activity was quantified as a baseline ratio relative to a standardized static chudan-no-kamae posture. Trial-level mixed-effects models were used to analyze these outcomes.

**Results:**

The results showed that no statistically significant between-group differences were detected in RT or false responses. In contrast, elite players demonstrated significantly greater peak acceleration and peak jerk than sub-elite players, indicating greater peak shinai acceleration and a more rapid change in acceleration during movement initiation. In addition, the odds of premature reactions were significantly lower in the elite group, whereas no statistically significant overall group effect was detected for pre-movement muscle activity.

**Discussion:**

Taken together, these findings suggest that differences in high-level expertise in Kendo counterattacks are reflected in movement characteristics immediately before and after movement initiation, rather than solely in the timing or correctness of response onset.

## 1 Introduction

The ability to rapidly select and execute appropriate reactions under time constraints is essential in many sports. In baseball and cricket, for example, achieving high batting performance requires precise swing timing and accurate bat control in response to pitches (1, 2). In interpersonal sports such as Kendo and boxing, the success of defensive actions and counterattacks against an opponent's attacks largely determines control of the exchange and ultimately the match outcome (3, 4). These forms of reactive behavior rely not only on mere physical quickness but also on the ability to initiate movement at the appropriate moment and to transition smoothly into subsequent actions in response to situational demands (5, 6).

The speed of reactions in sports is commonly evaluated using reaction time (RT), defined as the time from the presentation of an external stimulus to the initiation of a voluntary movement. Whole-body RT (WBRT), typically measured in response to a light stimulus, is widely used as an indicator of physical performance (7, 8). Indeed, previous research has shown that athletes generally exhibit shorter RTs than non-athletes (9). However, in offensive–defensive exchanges in interpersonal sports, a faster reaction is not necessarily better. Since there is a trade-off between speed and accuracy of reactions (10), excessively early reactions made before adequately interpreting an opponent's movements may result in false responses. Elite goalkeepers in soccer (11) and handball (12), for example, have been shown to delay the initiation of their reactions to maintain accuracy and response at a more appropriate moment. Similarly, in Kendo, skilled practitioners can maintain their stance—referred to as kamae—until just before initiating a counterattack (13). Taken together, these findings suggest that the timing of a reaction's initiation strongly influences performance in interpersonal sports, and that the manner in which this timing is regulated may vary depending on skill level.

In this study, we focus on counterattacks in Kendo, a sport in which offensive and defensive roles shift within a very short time frame. Kendo is a martial art in which practitioners strike with a bamboo sword (shinai), and similar to fencing, it requires them to accurately read an opponent's movements and to execute reactive actions based on appropriate preparatory states (14). In such exchanges, a series of processes—such as maintaining an appropriate preparatory state from the ready position until the initiation of the main movement, determining the timing of reaction initiation, and producing a sharp movement onset after initiation—are considered to influence the success of the reaction. In this study, we defined this sequence of processes collectively as reaction strategies. Reaction strategies represent the characteristic manner in which skilled athletes regulate reaction initiation, and their specific features may vary depending on the characteristics of the sport and situational demands. A previous study investigating high-level Kendo athletes reported that those with lower skill levels exhibited more fluctuations in shinai acceleration that exceeded a predefined threshold before the onset of the main movement during counterattacks (15). This phenomenon, defined as a premature reaction, represents an objectively detected early movement fluctuation and may be related to variations in the preparatory state prior to reaction initiation.

Pre-movement muscle activity is widely used to assess the preparatory state prior to reaction initiation (16, 17). Although Araki and Choshi suggested that a moderate level of pre-movement muscle activity facilitates rapid movement initiation (18), Zhang and colleagues reported that skilled tennis players exhibit lower levels of muscle activation than less-skilled players during sport-specific actions, reflecting more efficient movement control (19). In addition, excessive upper-limb muscle activity has been reported to delay reactions in Kendo as well (20). Therefore, clarifying the characteristics of pre-movement muscle activity and premature reactions prior to main movements is important to further understand reaction strategies in Kendo.

Furthermore, movement patterns after the onset of a reaction would also underpin the reaction strategies. The acceleration and jerk during the initial phase of movement are representative indicators of the sharpness of movement initiation (21). In combat sports (22) and racket sports (23), skilled athletes have been reported to exhibit sharper movement initiation specific to the demands of their sport. Although previous studies in Kendo have shown skill-level differences in RT—from the presentation of a stimulus to the onset of striking—and in movement time until strike completion (24), no studies have specifically examined the sharpness of movement initiation itself after reaction onset.

Based on these backgrounds, this study examined how differences in skill level influence reaction strategies in Kendo, by using conventional indicators of reaction. We attempted to quantify the premature reactions, pre-movement muscle activity prior to reaction initiation, and the sharpness of shinai movement initiation after the reaction begins. We recruited two groups of high-level university Kendo athletes—elite players and sub-elite players—to examine whether skill-level differences would be evident not only in standard RT and response accuracy but also in processes occurring before and after reaction initiation. Participants performed a reaction task of counterattack selection using a life-size video stimulus. Two counterattacks frequently observed in actual competition, Men-Kaeshi-Do and Kote-Kaeshi-Men, were adopted as the task conditions. Figure 1 illustrates the sequence of phases involved in each counterattack. To describe the actions used in the task, Kendo strike terminology is used in this study. A Men strike refers to an attack directed toward the head, a Kote strike refers to an attack directed toward the forearm near the wrist, and a Do strike refers to an attack directed toward the torso. These terms are used to describe both the opponent's offensive actions and the participants’ counterattacks throughout the whole manuscript. Group differences were then analyzed using a series of indices obtained before and after reaction initiation to examine whether skill-level differences were evident in these high-performance athletes’ reaction strategies.

**Figure 1:**
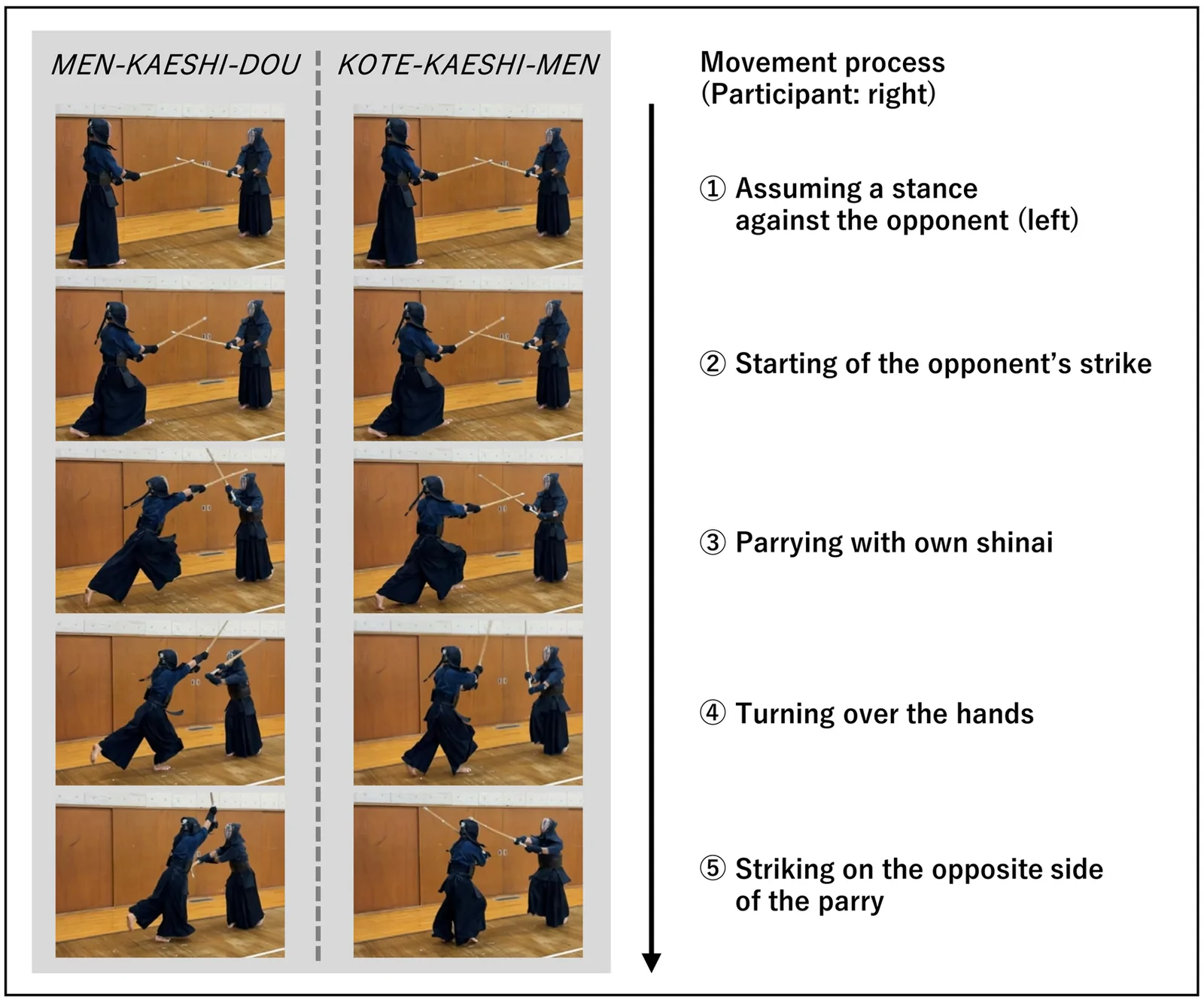
Movement phases of counterattacks in Kendo.

We hypothesized that skill-level differences would be more evident in the processes occurring before and after reaction initiation than in standard indices such as RT or response accuracy. In particular, elite players would exhibit a lower frequency of premature reactions and reduced pre-movement muscle activity than sub-elite players. We also hypothesized that elite players exhibit sharper movement initiation than sub-elite players during counterattacks.

## 2 Methods

### 2.1 Participants

A total of 18 male university Kendo athletes who belong to a collegiate Kendo club participated in this study. Participants were divided into an elite group (*n* = 9) and a sub-elite group (*n* = 9) based on whether they had experience competing as elite members in major university tournaments. Among the elite group, eight athletes had experience winning the All Japan Student Kendo Championship as team members. In the sub-elite group, all nine athletes had achieved top placements in regional tournaments, and four of them had earned top placements at national-level tournaments during high school. Details of age, height, body weight, and years of competitive experience for each group are presented in Table 1.

**Table 1:** Physical characteristics of the participants (mean ± SD).

Variables	Elite (*n* = 9)	Sub-elite (*n* = 9)	*p* value	Effect size
Age (years)	20.9 ± 0.6	19.6 ± 0.9	0.002	1.77
Height (cm)	172.0 ± 5.2	171.6 ± 5.6	0.864	0.08
Body Weight (kg)	71.6 ± 4.7	74.0 ± 6.4	0.369	0.44
Competition history (years)	15.0 ± 1.6	14.2 ± 1.3	0.778	0.54

Values are presented as mean ± SD. Effect sizes are expressed as Cohen's *d*. Elite, *n* = 9; sub-elite, *n* = 9.

This study was approved by the Research Ethics Committee of the Institute of Health and Sport Sciences, University of Tsukuba (approval number: Tai 022-129). All participants were informed of the purpose and procedures of the study, and written informed consent was obtained prior to participation. The experiment was conducted with full consideration for participant safety.

A comparison of physical characteristics between the two groups showed that the sub-elite group was significantly younger than the elite group (*p* = 0.002, *d* = 1.77). Eight of the nine elite athletes were upper-year students (third- or fourth-year), whereas eight of the nine sub-elite athletes were first- or second-year students.

### 2.2 Counterattack reaction task

A counterattack reaction task using video-based stimuli was conducted in this study (Figure 2). The reaction stimuli consisted of video clips recorded with four skilled Kendo practitioners (4th-dan holders) serving as opponents. The recordings were made to ensure that the striking motions reflected realistic offensive-defensive exchanges in actual Kendo matches (Figure 3). The duration of the initial shinai-raising movement was checked from the recorded videos, and no marked differences in movement duration were observed among the four opponents. The use of multiple opponents also allowed natural inter-opponent variability in striking movements to be retained rather than relying on a single standardized movement pattern. The videos were projected life-size onto a screen (approximately 300 cm wide × 230 cm high, Figure 2a) positioned in front of the participants (Figure 2b), with the projector (EB-X8, EPSON Inc, Figure 2c) mounted above them. The video resolution was 1920 × 1080 pixels and a frame rate of 30 fps. The experiment was conducted under near-darkened room conditions to ensure clear projection of the video stimuli, and the lighting conditions were kept consistent across all participants.

**Figure 2:**
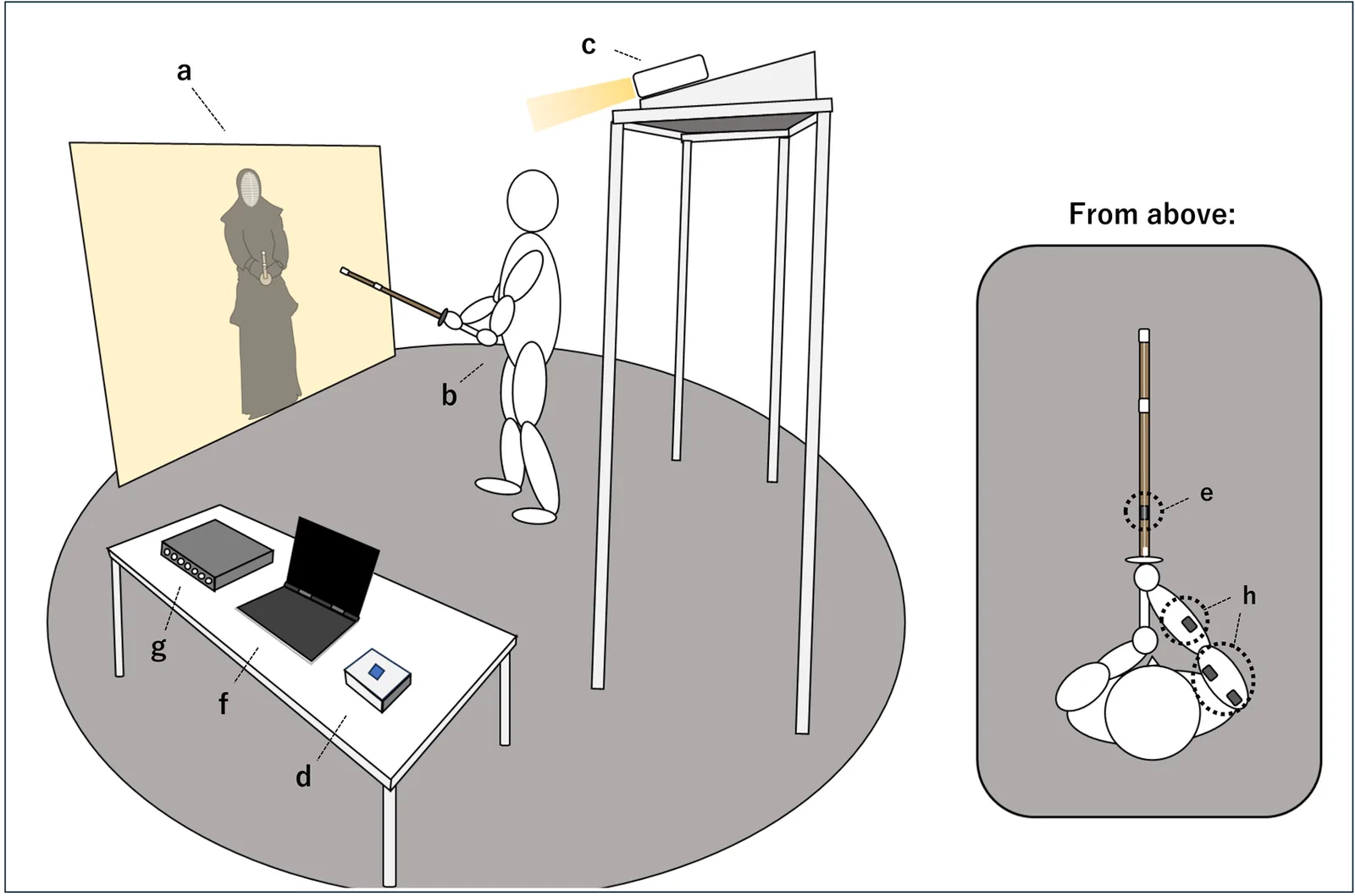
Experimental setup of the counterattack reaction task. **(a)** screen; **(b)** participant; **(c)** projector; **(d)** trigger switch; **(e)** accelerometer; **(f)** PC; **(g)** A/D converter; **(h)** active electrodes.

**Figure 3:**
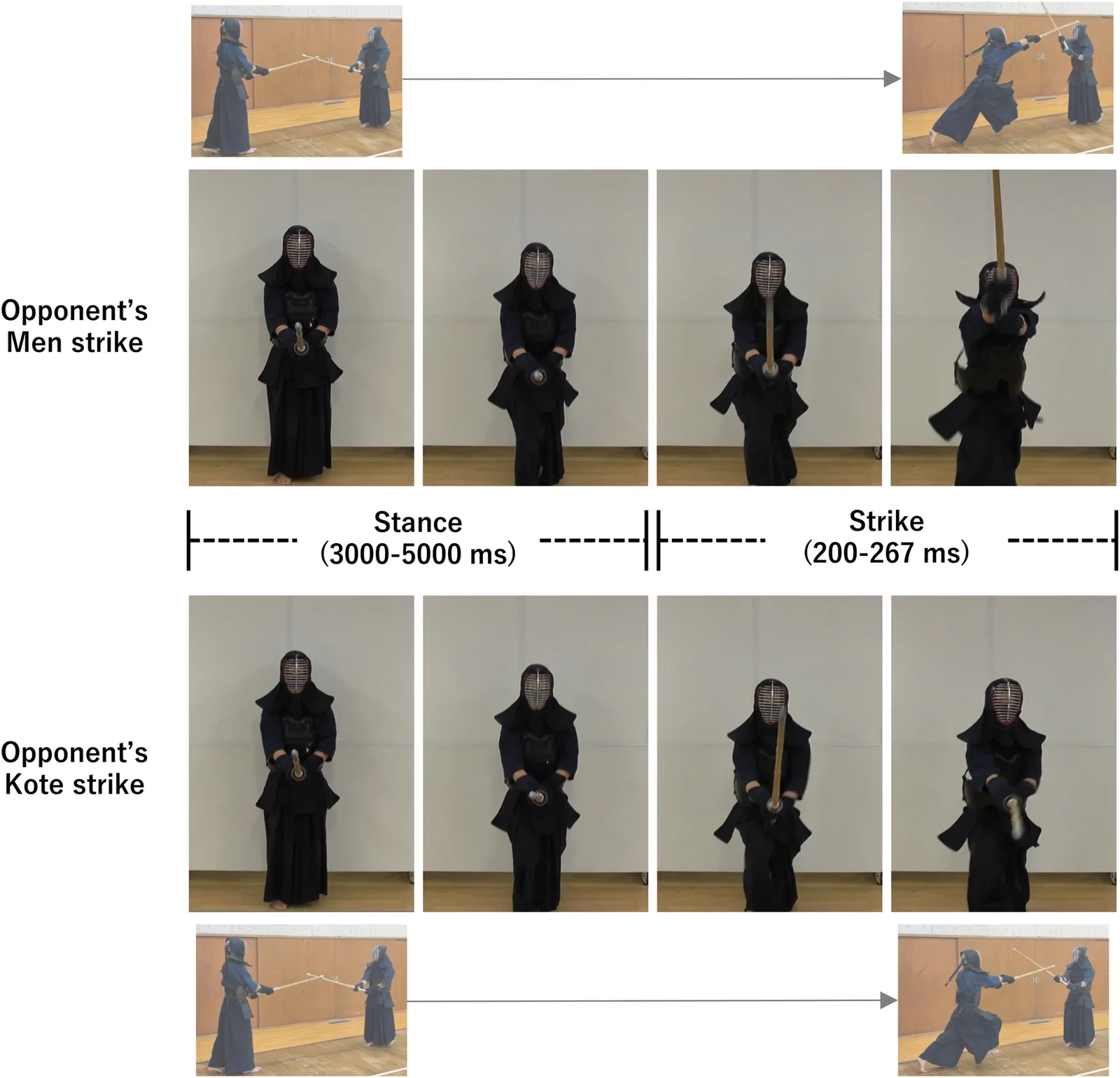
Striking actions of the opponent presented in the video stimuli. In the counterattack reaction task, the opponent began from a stance (left panel) and, after a randomized delay, initiated either a Men strike (upper right) or a Kote strike (lower right) toward the participant.

In the task, participants viewed video stimuli in which the opponent on the screen initiated either a straight Men strike or a right Kote strike (Figure 3). These striking motions were originally performed toward a virtual target positioned directly in front of the camera during filming. However, since the videos were projected at life-size onto the screen in front of the participant, the attacks were perceived as directed at the participant during the task.

Participants stood in a ready position with the tip of their right foot aligned 230 cm from the screen. This distance was selected since it approximated Issoku-itto-no-maai (25)—the fundamental striking distance in Kendo—when translated to the present experimental setup in which the opponent's actions were projected onto a flat screen. This positioning allowed the task to simulate the interpersonal distance at which a practitioner can reach the opponent with a single step forward.

When the tester pressed a trigger switch (Figure 2d), the video started, and after a delay of 3, 4, or 5 seconds, the opponent's shinai began to rise upward, initiating either a Men or a Kote strike (Figure 3). Each combination of opponent (4 individuals), delay timing (3 levels), and strike type (2 types) was presented once, resulting in 24 trials per participant. The presentation order of the 24 stimulus conditions was randomized using a PowerPoint macro before the experiment. The same randomized sequence was presented to all participants and was not re-randomized separately for each participant.

Participants were instructed to select and execute the appropriate counterattack for each stimulus. Specifically, when the opponent performed a Men strike, participants executed Men-Kaeshi-Do, in which the incoming Men strike is parried, and the opponent's right Do is struck. When the opponent performed a Kote strike, participants executed Kote-Kaeshi-Men, in which the Kote strike is parried, and the opponent's Men is struck. These two counterattacks were selected since they involve similar movement patterns and are frequently used in actual Kendo.

Since the task required participants to respond to video stimuli, they could not physically block the opponent's shinai or make actual contact strikes. However, participants were instructed to perform the imagined counterattack actions as realistically as possible. Participants were allowed to take rest breaks between trials as needed to avoid fatigue.

### 2.3 Data processing

#### 2.3.1 Video data of the opponent

To determine the RT in the counterattack reaction task, the onset of the opponent's movement in the video was determined by consensus among three highly skilled Kendo practitioners (two 8th-dan holders and one 7th-dan holder). The onset was determined frame-by-frame (30 fps) and defined as the moment at which the opponent's shinai began to move upward to initiate the strike.

#### 2.3.2 Accelerometer data

To obtain acceleration and jerk data, an accelerometer (MA3-10Ac, Micro Stone Inc.; Figure 2e) was attached to the participant's shinai. The sensitivity of the sensor was 20 mV per m/s^2^ (±10%). To evaluate the sharpness of movement initiation in shinai handling, the vertical (z-axis) acceleration was recorded at a sampling rate of 1000 Hz and digitized via an A/D converter (1401, CED Inc.; Figure 2g) into data analysis software (Spike2, CED Inc.; Figure 2f). When the video-start switch was pressed, a trigger signal was simultaneously sent to the A/D converter to synchronize the video and acceleration data. The acceleration signal was low-pass filtered using a fourth-order Butterworth filter with a cutoff frequency of 80 Hz. Bidirectional filtering was applied to avoid phase distortion. The relatively high cutoff frequency was selected to attenuate high-frequency noise while preserving the rapid transient characteristics of the shinai acceleration signal, as excessively low cutoff frequencies can attenuate peak acceleration during rapid sports movements (26), and an 80-Hz cutoff has been used for impact-related sports acceleration data based on residual analysis (27). The filtered acceleration signal was used for all subsequent acceleration-based analyses, including participant movement onset detection, premature reaction detection, peak acceleration, and jerk calculation. Jerk was calculated by numerically differentiating the filtered acceleration signal with respect to time at the original sampling rate of 1000 Hz. No additional filtering or smoothing was applied after differentiation. This processing order was used to reduce the amplification of high-frequency noise during numerical differentiation (28, 29).

The onset of the participant's movement was determined based on the acquired acceleration data. Movement onset was defined as the moment at which the shinai began to move vertically, in the direction corresponding to the z-axis of the accelerometer, from the ready position, operationalized as the time point when the change in acceleration exceeded the baseline mean by 3 SD in the filtered acceleration signal (30, 31). The baseline mean and SD were calculated from the acceleration signal between the start of the video and 500 ms before the opponent's movement onset. The first acceleration peak corresponding to the initial upward movement of the counterattack and the rising phase leading to this peak were visually identified. Participant movement onset was then determined within this rising phase.

Premature reactions were identified separately from the participant movement onset used to calculate RT. A premature reaction was defined as a transient acceleration fluctuation occurring between 500 ms before the opponent's movement onset and the participant's movement onset that exceeded the same baseline mean + 3 SD threshold. The acceleration waveform was visually inspected to confirm that the threshold crossing represented a transient fluctuation that subsequently returned toward the baseline level rather than the rising phase of the counterattack itself. A trial was classified as containing a premature reaction when at least one such threshold crossing was observed within this interval. The 500-ms boundary was selected because preparatory body movement of the opponent was visually observable for approximately 500 ms before the shinai movement defined as opponent movement onset; extending the interval further backward could therefore include fluctuations unrelated to the opponent's attack-related movement. Thus, a premature threshold crossing did not replace the participant movement onset used for RT calculation. This interval is hereafter referred to as the pre-strike phase.

#### 2.3.3 EMG data

Surface electromyography was recorded using a wired EMG system (Bagnoli-2 EMG System, Delsys Inc.) and active electrodes (DE-2.1, Delsys Inc.; Figure 2h). The target muscles were those involved in raising the shinai during counterattacks, and all electrodes were placed on the participant's right side. The recorded muscles were the anterior deltoid (AD), biceps brachii (BB), and flexor carpi radialis (FCR).

EMG signals were digitized via the A/D converter at a sampling rate of 1000 Hz and stored in the data analysis software alongside the synchronized video and acceleration data. A band-pass filter (10–350 Hz) was applied during EMG acquisition to remove noise. Before electrode placement, the skin surface was cleaned with alcohol to reduce impedance. No additional rectification, smoothing, or filtering was applied before RMS calculation; RMS values were calculated directly from the band-pass-filtered EMG signals.

Before the counterattack reaction task, a reference EMG measurement was obtained while each participant maintained a standardized static chudan-no-kamae posture while holding the shinai. A 500-ms segment without apparent movement fluctuation or signal noise was selected by visual inspection of the synchronized acceleration and EMG waveforms, and the RMS value obtained from this segment was defined as the baseline RMS for each muscle. The chudan-no-kamae posture was selected as a task-specific reference condition to quantify pre-movement muscle activity relative to a consistent Kendo-specific posture rather than to maximal activation capacity (32, 33). For each trial, pre-movement EMG activity was quantified as the RMS amplitude during the 200 ms immediately preceding the participant's movement onset. This value was normalized to the corresponding muscle-specific baseline RMS and expressed as a baseline ratio (pre-movement RMS/baseline RMS), with a value of 1.0 representing the baseline level.

### 2.4 Outcome measures

#### 2.4.1 Reaction time (RT)

To evaluate the quickness of movement initiation, the reaction time for counterattacks in Kendo (RT) was calculated. RT was defined as the time interval between the onset of the opponent's movement and the onset of the participant's movement (Figure 4). For each participant, the mean RT across all trials was used for analysis.

**Figure 4:**
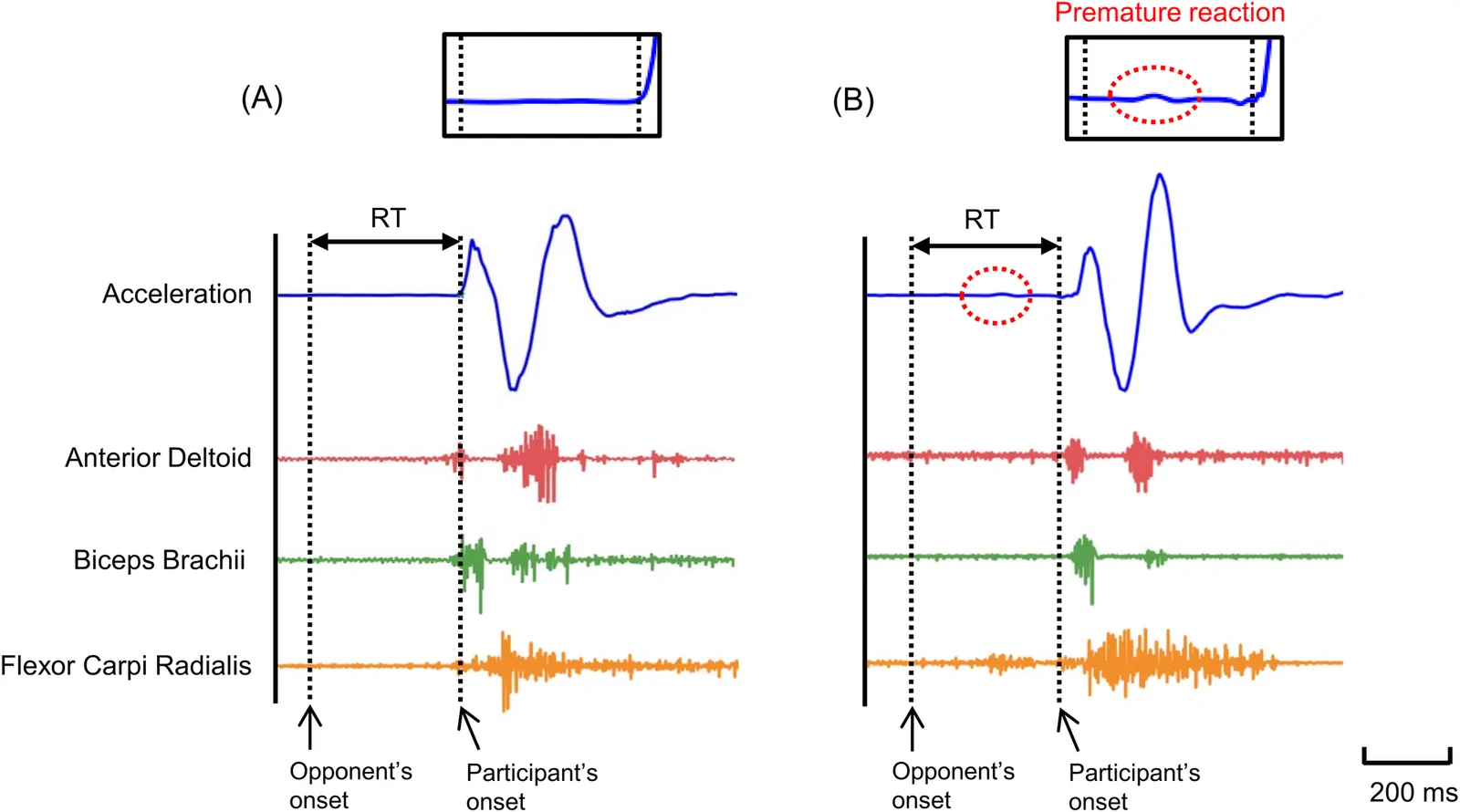
Representative examples of acceleration and EMG data. **(A)** Representative acceleration and upper-limb EMG signals during a normal counterattack reaction. **(B)** An example in which a premature reaction occurred (highlighted by the red dashed box). RT was calculated as the interval between the opponent's movement onset and the participant's movement onset, defined as the time point at which the participant's shinai acceleration exceeded the threshold. The participant's movement onset was identified from the rising phase leading to the first acceleration peak using the baseline mean + 3 SD threshold. Premature reactions were identified separately from the participant's movement onset as transient acceleration fluctuations exceeding the same threshold before movement onset.

#### 2.4.2 False responses

To evaluate response selection accuracy, false responses were identified for each trial. In this task, a false response was defined as an incorrect selection of a counterattack, such as executing Kote-Kaeshi-Men in response to a Men strike presented in the video stimulus. Participants were instructed to report any instances in which they recognized that an incorrect response had been selected. A trial was classified as a false response when an incorrect counterattack was identified either by the participant's self-report or by visual observation by the experimenter.

#### 2.4.3 Peak acceleration and peak jerk

To evaluate the sharpness of movement initiation in shinai handling, the peak value of vertical acceleration and the peak value of jerk (the rate of change in acceleration) were calculated. Peak jerk was identified within the period from the participant's movement onset to the time of peak acceleration.

#### 2.4.4 Premature reactions

To evaluate premature reactions, transient acceleration fluctuations occurring between 500 ms before the opponent's movement onset and the participant's movement onset were identified. As described above, a premature reaction was defined as a transient acceleration fluctuation that exceeded the baseline mean + 3 SD threshold and subsequently returned toward the baseline level before the participant's movement onset. Premature reactions were identified separately from the participant movement onset used to calculate RT. Each trial was classified according to the presence or absence of a premature reaction.

#### 2.4.5 Pre-movement muscle activity (baseline ratios)

Pre-movement muscle activity for each muscle was calculated from the surface EMG data (Figure 4). The analysis window was defined as the 200 ms period immediately before the onset of the participant's movement. Root mean square (RMS) values were calculated for each muscle, and each RMS value was normalized to the muscle-specific baseline RMS obtained during the standardized static chudan-no-kamae posture to yield a baseline ratio. A baseline ratio of 1.0 represented the baseline level. Baseline ratios were calculated separately for each muscle.

### 2.5 Statistical analysis

RT, peak acceleration, and peak jerk were analyzed as trial-level continuous outcomes using linear mixed-effects models (LMMs). Each model included group, opponent, attack type, stimulus timing, and trial order as fixed effects, with participant included as a random intercept. Trial order was entered as a centered continuous variable. False responses and premature reactions were analyzed as trial-level binary outcomes using generalized linear mixed-effects models (GLMMs) with a binomial distribution and logit link function. These models included the same fixed effects and a participant-level random intercept.

Pre-movement EMG activity was analyzed using an integrated LMM in which the baseline ratios for FCR, BB, and AD were retained as separate muscle-specific observations. Baseline ratios were log-transformed before analysis. The model included group, muscle, opponent, attack type, stimulus timing, and trial order as fixed effects. Muscle-specific participant-level random effects and a trial-level random intercept were included to account for the non-independence of repeated muscle-specific observations within participants and trials, and thus the hierarchical structure of the EMG data (34, 35). A model including a group × muscle interaction was also evaluated to assess whether the group effect differed among muscles. The additive and interaction models were compared using a likelihood-ratio test after fitting with maximum likelihood, and the additive model was retained for the final model.

To assess the potential influence of the between-group difference in age, age-adjusted sensitivity analyses were conducted for all outcomes by adding age as an additional fixed-effect covariate to the corresponding model.

False response rate and premature reaction rate were calculated as the number of events divided by the total number of trials and were used primarily for descriptive statistics and graphical presentation. The significance level was set at *p* < 0.05 for all statistical analyses. Given the relatively small sample size and the evaluation of multiple outcome measures without formal adjustment for multiplicity, individual p-values were interpreted cautiously and considered together with effect estimates and 95% confidence intervals. Statistical analyses were performed using Python (version 3.10.19; statsmodels, version 0.14.6) and R (version 4.6.1; lme4, version 2.0.6).

## 3 Results

### 3.1 Reaction time and false responses

Descriptive statistics and model-based group comparisons for reaction time (RT) and false responses in the counterattack reaction task are shown in Table 2. Individual participant data, together with the model-adjusted group estimates and 95% CIs, are shown in Figure 5. The LMM revealed no statistically significant between-group difference in RT (estimated difference [elite−sub-elite] = −4.2 ms, 95% CI = −38.8 to 30.4 ms, *p* = 0.810).

**Table 2:** Model-adjusted estimates and group comparisons for reaction time (RT) and false responses.

Outcome	Elite: adjusted estimate (95% CI)	Sub-elite: adjusted estimate (95% CI)	Group effect (95% CI)	*p* value
RT (ms)	386.8 (362.3, 411.3)	391.0 (366.5, 415.5)	Difference = −4.19 (−38.8, 30.4)	0.810
False response (%)	5.9 (2.6, 13.1)	4.6 (1.8, 11.2)	OR = 1.33 (0.37, 4.76)	0.658

Adjusted estimates are model-based estimates derived from the trial-level mixed-effects models. For RT, adjusted estimates are presented as adjusted means, and the group effect is expressed as the estimated mean difference (elite−sub-elite). For false responses, adjusted estimates are presented as adjusted probabilities, and the group effect is expressed as an odds ratio (elite vs. sub-elite). All 432 trials from 18 participants (*n* = 9 per group) were included in the analyses.

CI, confidence interval; OR, odds ratio; RT, reaction time.

**Figure 5:**
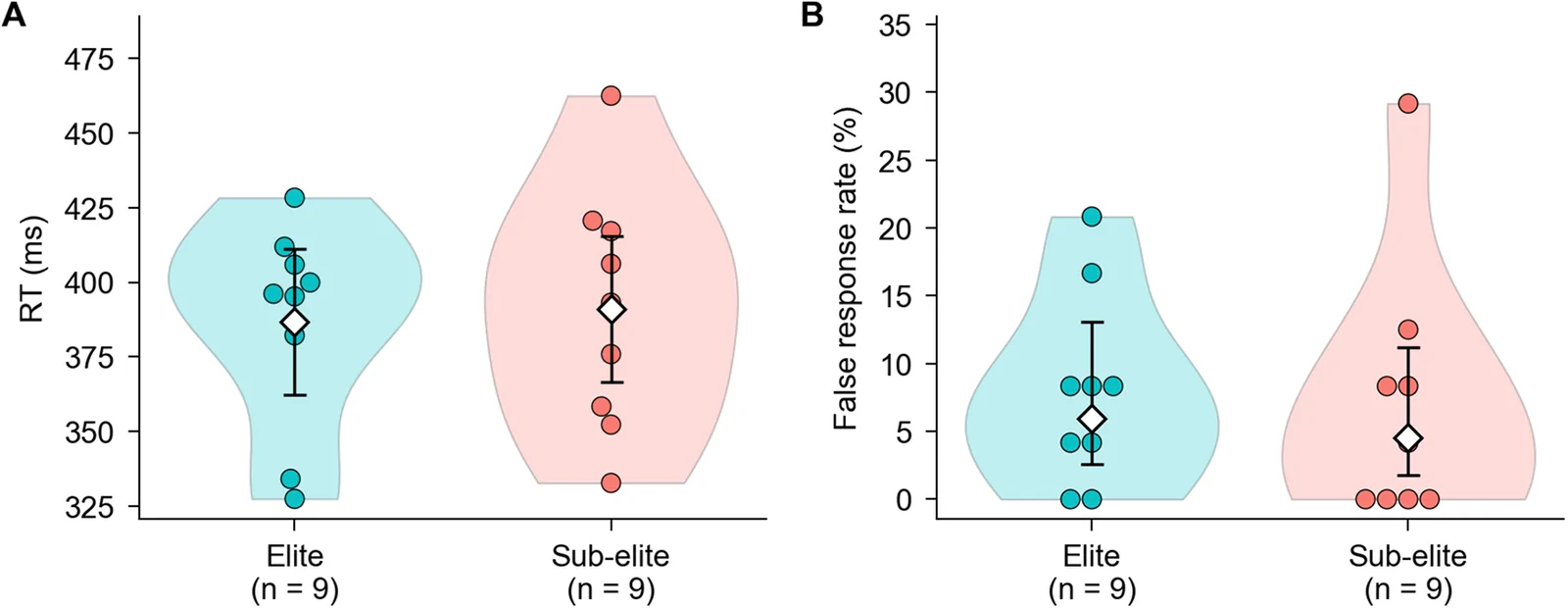
Violin plots of reaction time (RT) and false response rate for each group. **(A)** Reaction time (RT). **(B)** False response rate. Individual points represent participant-level values. Open diamonds indicate model-adjusted group estimates, and error bars indicate 95% confidence intervals. For false responses, participant-level points represent the percentage of trials with a false response.

For false responses, the binomial GLMM with a logit link function revealed no statistically significant difference in the odds of a false response between the two groups (OR [elite vs. sub-elite] = 1.33, 95% CI = 0.37–4.76, *p* = 0.658). The age-adjusted sensitivity analyses did not materially alter the direction or overall interpretation of the group effects for either RT or false responses (Supplementary Table 1).

### 3.2 Peak acceleration and peak jerk

The peak acceleration and peak jerk from reaction onset to the first acceleration peak, together with the model-adjusted group estimates and 95% CIs, are shown in Table 3 and Figure 6. Sensor output values (V) were converted using the sensor sensitivity of 20 mV per m/s^2^, and peak acceleration and peak jerk were expressed in units of m/s^2^ and m/s^3^, respectively.

**Table 3:** Model-adjusted estimates and group comparisons for peak acceleration and peak jerk.

Variables	Elite: adjusted estimate (95% CI)	Sub-elite: adjusted estimate (95% CI)	Group effect (95% CI)	*p* value
Peak acceleration (m/s^2^)	23.1 (20.3, 25.9)	15.8 (13.0, 18.6)	Difference = 7.3 (3.4, 11.2)	< 0.001
Peak jerk (m/s^3^)	758.2 (647.2, 869.2)	456.9 (345.9, 568.0)	Difference = 301.3 (144.3, 458.3)	< 0.001

Adjusted estimates are model-based estimates derived from the trial-level linear mixed-effects models. Adjusted estimates are presented as adjusted means, and the group effect is the estimated mean difference (elite−sub-elite). All 432 trials from 18 participants (*n* = 9 per group) were included in the analyses.

CI, confidence interval.

**Figure 6:**
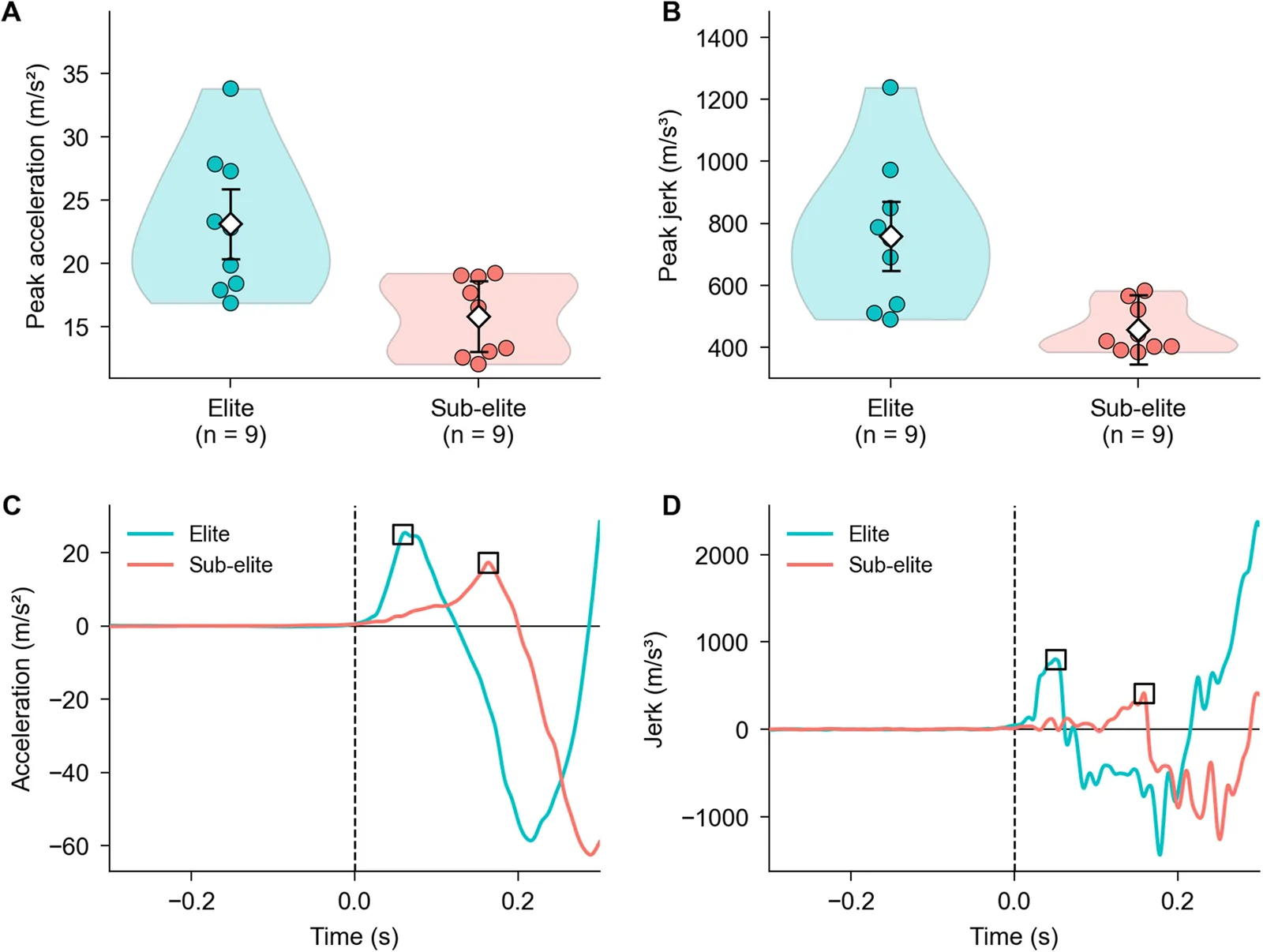
Violin plots and representative waveform examples for peak acceleration and peak jerk. **(A)** Violin plots for peak acceleration in each group. **(B)** Violin plots for peak jerk in each group. **(C)** Representative acceleration waveforms from each group. **(D)** Representative jerk waveforms from each group. Individual points represent participant-level values. Open diamonds in panels (**A,B**) indicate model-adjusted group estimates, and error bars indicate 95% confidence intervals. Open squares in panels (**C,D**) indicate the peak values. The waveforms in panels (**C,D**) are representative individual examples and do not represent group-averaged waveforms.

The LMM revealed that peak acceleration was significantly greater in the elite group than in the sub-elite group (estimated difference [elite−sub-elite] = 7.3 m/s^2^, 95% CI = 3.4 to 11.2 m/s^2^, *p* < 0.01). Peak jerk was also significantly greater in the elite group than in the sub-elite group (estimated difference [elite−sub-elite] = 301.3 m/s^3^, 95% CI = 144.3 to 458.3 m/s^3^, *p* < 0.01). The age-adjusted sensitivity analyses did not materially alter the direction or overall interpretation of the group effects for either peak acceleration or peak jerk (Supplementary Table 1).

### 3.3 Premature reactions and pre-movement muscle activity

Descriptive statistics and model-based group comparisons for premature reactions and pre-movement muscle activity are shown in Table 4. Individual participant data, together with the model-adjusted group estimates and 95% CIs, are shown in Figure 7. For pre-movement muscle activity, participant-level data are presented as geometric means, and model-adjusted estimates are presented as geometric means derived from the log-transformed LMM.

**Table 4:** Model-adjusted estimates and group comparisons for premature reactions and pre-movement muscle activity.

Variables	Elite: adjusted estimate (95% CI)	Sub-elite: adjusted estimate (95% CI)	Group effect (95% CI)	*p* value
Premature reaction (%)	7.1 (4.0, 12.3)	18.6 (12.5–26.8)	OR = 0.37 (0.15, 0.70)	0.004
AD baseline ratio	1.38 (1.09, 1.75)	1.68 (1.33, 2.13)	—	—
BB baseline ratio	1.12 (0.88, 1.41)	1.36 (1.07, 1.72)	—	—
FCR baseline ratio	1.63 (1.32, 2.02)	1.99 (1.61, 2.46)	—	—
Overall baseline ratio	1.36 (1.16, 1.59)	1.66 (1.42, 1.94)	Raito = 0.82 (0.66, 1.02)	0.078

Adjusted estimates are model-based estimates derived from the trial-level mixed-effects models. For premature reactions, adjusted estimates are presented as adjusted probabilities, and the group effect is the odds ratio (elite vs. sub-elite). For pre-movement EMG activity, adjusted estimates are presented as geometric means back-transformed from the log-transformed linear mixed-effects model. The Overall estimate represents the adjusted geometric mean obtained by equally weighted marginalization across FCR, BB, and AD. The EMG group effect is expressed as the elite-to-sub-elite ratio estimated across the three muscles in the integrated additive model. Muscle-specific group effects were not estimated separately and are therefore indicated by em dashes (—). A baseline ratio of 1.0 represents the baseline level.

AD, anterior deltoid; BB, biceps brachii; CI, confidence interval; FCR, flexor carpi radialis; OR, odds ratio.

**Figure 7:**
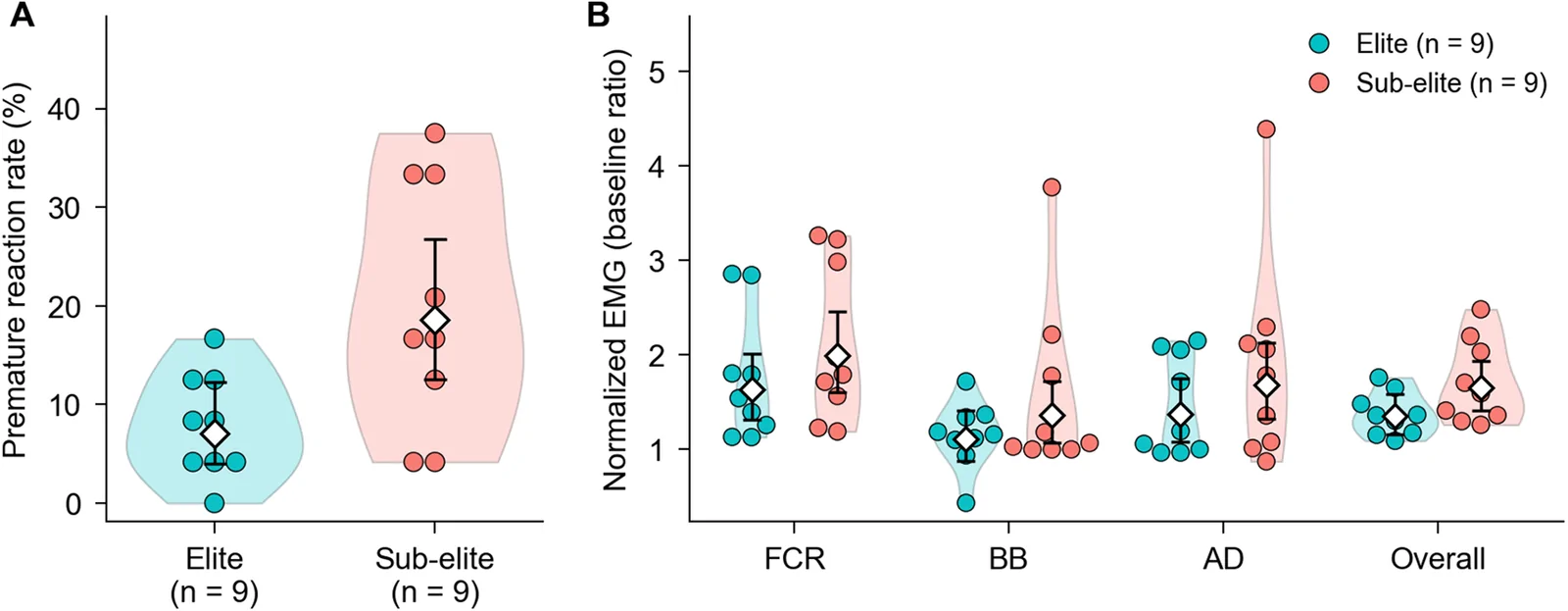
Violin plots of premature reaction rate and pre-movement muscle activity for each group. **(A)** Premature reaction rate. **(B)** Pre-movement muscle activity expressed as the baseline ratio during the 200 ms immediately prior to movement onset for FCR, BB, AD, and Overall. Individual points represent participant-level values. For premature reactions, participant-level points represent the percentage of trials with a premature reaction. For EMG, participant-level points represent geometric mean baseline ratios; Overall represents the geometric mean across FCR, BB, and AD for each participant. Open diamonds indicate model-adjusted estimates, and error bars indicate 95% confidence intervals. For EMG, the adjusted estimates are geometric means back-transformed from the log-transformed LMM; the Overall estimate was obtained by equally weighted marginalization across FCR, BB, and AD.

For premature reactions, binomial GLMM revealed a significant group difference in the odds of occurrence. Specifically, the odds of premature reactions were significantly lower in the elite group than in the sub-elite group (OR [elite vs. sub-elite] = 0.37, 95% CI = 0.15–0.70, *p* < 0.01).

For pre-movement muscle activity during the 200 ms immediately preceding reaction initiation, baseline ratios for FCR, BB, and AD were analyzed together using an integrated LMM. There was no evidence of a group × muscle interaction (likelihood-ratio *χ*^2^(2) = 0.078, *p* = 0.962); therefore, the additive model was retained as the final model. In this model, no statistically significant overall group effect was detected for pre-movement muscle activity (ratio [elite/sub-elite] = 0.82, 95% CI = 0.66–1.02, *p* = 0.078). The age-adjusted sensitivity analyses did not materially alter the direction or overall interpretation of the group effects for either premature reactions or pre-movement muscle activity (Supplementary Table 1).

## 4 Discussion

### 4.1 Reaction time and response accuracy

To examine reaction speed and response accuracy in the counterattack reaction task, reaction time (RT) and false responses were evaluated. No statistically significant between-group differences were detected for either measure (Table 2, Figure 5). Given the competitive background of the participants, both the elite and sub-elite groups can be considered to comprise highly skilled athletes, and the relatively high skill level of both groups may have limited the between-group contrast in the timing or accuracy of counterattack initiation. However, the absence of statistically significant differences may also reflect the limited precision associated with the small sample size.

Unlike conventional reaction tasks that rely on simple sensory stimuli, the present task required participants to react to a video of an opponent, which is more representative of competitive Kendo. Under such conditions, it is difficult to identify a single, discrete visual event that serves as an effective trigger for a reaction. For the purpose of quantifying RT, the moment at which the opponent's shinai began to rise was operationally defined as the stimulus onset, corresponding to the “stimulus presentation” commonly used in previous RT studies. However, since the shinai continued to move toward the strike after this initial rise, this moment may not have served as the decisive trigger for initiating a response.

When compared with choice RTs reported in previous studies using shinai movements in response to simple light stimuli (36), the RTs observed in the present study (approximately 400 ms) were relatively long. This finding may suggest that participants continued to process visual information closer to the actual strike before initiating the selected counterattack. As illustrated in Figure 3, the opponent's intended strike is difficult to infer at the moment the shinai first begins to rise. Accordingly, the RTs calculated in this study may reflect the processing of dynamic, continuous visual information rather than a simple reaction to a discrete sensory cue.

Taken together, the absence of statistically significant group differences in RT and false responses does not provide evidence that the groups differed in the balance between reaction speed and response accuracy. One possible interpretation is that athletes in both groups may have prioritized response accuracy by delaying the initiation of the response until sufficient visual information was available. Hinz and colleagues reported that elite athletes are able to spend more time “waiting and seeing” by flexibly integrating top-down and bottom-up control during decision-making (12). Although their study focused on handball, Kendo likewise requires athletes to respond rapidly to an opponent's actions, suggesting that such a reaction strategy could also be advantageous in this context.

### 4.2 Sharpness of shinai movement initiation

Acceleration describes the rate of change in velocity, whereas jerk is defined as the time derivative of acceleration. Therefore, when evaluating the initiation of movements that change the position of the shinai, using higher-order derivatives such as acceleration and jerk provides a more detailed characterization of the sharpness of this initial movement phase.

Jerk has also been widely used as an index of movement smoothness (37). In general, larger jerk values are interpreted as reflecting abrupt or poorly coordinated movements, whereas smaller values indicate smoother motor control. In the present study, however, participants were required to initiate counter techniques explosively from an almost static posture. Under these task demands, the peak values of acceleration and jerk are better interpreted as indicators of the sharpness of the transition from a static to a dynamic state, rather than as measures of movement smoothness.

Consistent with this interpretation, the results demonstrated that the elite group exhibited significantly greater peak acceleration and peak jerk during the initial phase of shinai handling following reaction onset compared with the sub-elite group (Table 3, Figure 6). These findings indicate that the elite group exhibited greater peak shinai acceleration and a more rapid change in acceleration immediately after the onset of the counter motion. Similar skill-related differences in the sharpness of movement initiation have been reported in other sports. For example, Goethel and colleagues showed that elite karate athletes exhibited greater peak acceleration during punching movements in response to simple light stimuli than sub-elite athletes (22). Likewise, Masu and Otsuka reported that higher-level badminton players demonstrated larger increases in muscle activation amplitude and shorter movement times during both simple and choice reaction tasks (23).

One possible interpretation of the greater peak acceleration and peak jerk in the elite group is that the selected counterattack was more fully specified at the time of movement onset, allowing the subsequent shinai movement to develop more rapidly. Importantly, movement onset does not necessarily indicate that perceptual processing or action regulation has been fully completed. Previous studies of interceptive actions have shown that perceptual information continues to contribute to the regulation and refinement of ongoing movements after movement initiation. For example, Le Runigo and colleagues reported that tennis experts adapted an ongoing interceptive movement to unexpected changes in stimulus velocity with a shorter visuomotor delay than non-experts, suggesting more efficient perception–action coupling with expertise (38). Similarly, research on cricket batting has demonstrated that skilled performers use information available at different stages of an unfolding action to guide and refine interceptive movements (39).

From this perspective, the present combination of no statistically significant between-group difference in RT and greater post-onset peak acceleration and peak jerk in the elite group may be relevant. Although participants in the two groups initiated their movements at broadly similar times relative to the defined opponent movement onset, the subsequent development of shinai movement differed between groups. One possibility is that the elite athletes had more fully specified the selected counterattack by the time movement onset was detected, whereas in the sub-elite athletes, perceptual processing and action regulation may have continued to refine the selected response after movement onset. Such continued regulation could contribute to a more gradual development of acceleration and jerk. The representative waveforms shown in Figure 6 are consistent with this possibility in illustrating different temporal patterns of acceleration and jerk development after movement onset; however, these traces represent individual examples and should not be interpreted as group-level evidence of the underlying decision process.

Accordingly, the greater peak acceleration and peak jerk observed in the elite group indicate a skill-related difference in the kinematic characteristics of shinai movement after counterattack initiation. Together with the absence of a statistically significant group difference in RT, these findings raise the possibility that skill-related differences may involve not only when a movement is initiated, but also how fully the selected action is specified and how the movement develops after onset. This interpretation remains tentative because perceptual processing, decision confidence, and online action selection were not directly measured in the present study.

### 4.3 Premature reactions and pre-movement muscle activity

Focusing on how participants prepared for counterattacks, we examined premature reactions and pre-movement muscle activity in the FCR, BB, and AD. The odds of premature reactions were significantly lower in the elite group than in the sub-elite group, whereas no statistically significant overall group effect was detected for pre-movement muscle activity in the integrated LMM (Table 4, Figure 7). One factor potentially underlying the group difference in premature reactions is the opponent's preparatory movements that preceded the overt striking action and served as the reaction stimulus in the present task. As described earlier, the onset of the opponent's movement was operationally defined as the moment at which the shinai began to rise. In actual Kendo striking actions, however, subtle preparatory movements typically occur prior to this point. For example, in practical Men strikes, forward movement initiated by the right leg precedes the upward motion of the shinai, and skilled practitioners are instructed to withhold shinai movement until immediately before the strike (24). It is reasonable to assume that the experienced Kendo practitioners who served as opponents in the video stimuli exhibited similar movement patterns. Accordingly, the premature reactions—particularly those observed more frequently in the sub-elite group— may have been associated with responses to these subtle preparatory movements occurring just before the shinai began to rise.

From the perspective of the speed–accuracy trade-off, such early movement fluctuations may occur before sufficient information for the subsequent counterattack has been obtained, although their functional consequences cannot be determined directly from the present acceleration data. In Kendo instruction, the expression “do not break your stance” is commonly used to emphasize the importance of maintaining a stable, ready posture until immediately before initiating the main action. Since premature reactions in the present study were detected as transient deviations from the acceleration baseline associated with the ready stance, the higher frequency of premature reactions in the sub-elite group may reflect greater fluctuations in the preparatory state of the shinai before counterattack initiation. This interpretation concerns the objectively detected movement fluctuations and does not necessarily imply that every premature reaction represented an inappropriate or maladaptive response.

The analysis of pre-movement muscle activity further highlights the role of preparatory tension prior to reaction initiation. Many participants exhibited higher muscle activity during the 200-ms interval preceding movement onset than during the ready stance (Figure 7b). A moderate level of preparatory tension has been reported to facilitate rapid movement initiation (18), and the present findings are consistent with this notion. In addition, increases in muscle activity have been observed under psychological pressure (40, 41), suggesting that participants may have experienced some degree of task-related tension while responding to the opponent depicted in the video stimuli. Although psychological pressure was not directly measured in the present study, this possibility suggests that even two-dimensional video-based stimuli may reproduce some perceptual characteristics of actual combat situations.

Importantly, however, the integrated LMM did not detect a statistically significant overall group effect in pre-movement EMG activity, and there was no evidence that the group effect differed across FCR, BB, and AD. Although the group differences across the three muscles showed a generally consistent direction, the present results were insufficient to establish a statistically significant difference in pre-movement muscle activity between the elite and sub-elite groups. This may partly reflect the relatively small sample size, which may have limited the precision of these estimates and the ability to detect modest between-group differences. Accordingly, the absence of statistical significance should not be interpreted as evidence of equivalent pre-movement muscle activity between the groups.

The lower odds of premature reactions in the elite group may also be considered in relation to neural processes involved in suppressing or regulating unnecessary movements, although such mechanisms were not directly assessed in the present study. Studies of response inhibition commonly employ Go/NoGo tasks, in which participants are required to execute a response to Go stimuli while withholding responses to NoGo stimuli. The P300 component elicited during Go/NoGo tasks has been shown to reflect cognitive and motor inhibitory processes (42). In a Kendo-related study, Kawai and colleagues reported that skilled practitioners exhibited significantly larger P300 amplitudes at the parietal site (Pz) during NoGo trials than less-skilled practitioners, suggesting greater allocation of attentional resources to stimulus discrimination and inhibitory control (43). These findings provide one possible framework for interpreting the lower frequency of premature reactions in the elite group. However, inhibitory control, attentional allocation, and neural activity were not directly measured in the present study; therefore, the lower odds of premature reactions in the elite group should not be interpreted as direct evidence of superior inhibitory function. Future studies combining movement-based measures with direct neurophysiological or cognitive assessments would be required to examine this possibility.

### 4.4 Limitation

First, the sample size was relatively small, reflecting the limited availability of university Kendo athletes meeting the criteria for the elite group. Although the small number of participants may have limited the precision of the effect estimates, the trial-level mixed-effects models allowed the repeated-measures structure of the data to be appropriately accounted for while making use of all available trial-level observations. Second, the elite group was significantly older than the sub-elite group, and age, academic year, and competitive experience were closely related to group membership. Although age-adjusted sensitivity analyses did not materially alter the direction or overall interpretation of the principal group effects (Supplementary Table 1), the small sample size precluded complete separation of the effects of age and competitive level. Finally, interpretations involving inhibitory control, perceptual processing, decision-making, or online action selection remain tentative because these processes were not directly measured. Future studies with larger samples and more ecologically valid interpersonal tasks, together with whole-body kinematic, neurophysiological, and cognitive assessments, are needed to clarify the mechanisms underlying the present findings.

## 5 Conclusion

The purpose of this study was to clarify skill-level differences in reaction strategies during a counterattack reaction task in university Kendo players who competed at a comparably high performance level. No statistically significant between-group differences were detected in reaction time (RT) or response selection accuracy. However, clear group differences were observed in other aspects of counterattack preparation and execution. Specifically, the elite group showed lower odds of premature reactions and greater peak acceleration and peak jerk during the initial phase of shinai movement than the sub-elite group. In contrast, no statistically significant overall group effect was detected for pre-movement muscle activity.

These findings indicate that skill-level differences were evident both in the occurrence of premature movement fluctuations before counterattack initiation and in the kinematic development of the shinai movement after initiation. From a broader perspective, the present study is significant in that it elucidates skill-level differences in the execution of counterattacks in Kendo from physiological and kinematic perspectives, while also demonstrating that the absence of group differences in RT does not preclude differences in movement characteristics before and after movement initiation.

Future research should expand the participant population and experimental settings and incorporate more comprehensive assessments, including whole-body kinematics and neural activity, to further generalize the present findings and explore their practical applicability.

## Data Availability

The raw data supporting the conclusions of this article will be made available by the authors, without undue reservation.
