# Supplementary material for: Reaction strategies before and after the initiation of counterattacks in elite and sub-elite Kendo athletes
Source: Front Sports Act Living. 2026 Oct 21;8:1947221. doi: 10.3389/fspor.2026.1947221 (PMC13731267; doi:10.3389/fspor.2026.1947221)
Supplement: Supplementary file 1 [file Table1.docx]

Supplementary Material

Supplementary table 1. Age-adjusted estimates and between-group effects for behavioral, kinematic, and electromyographic outcomes

| Variables | Elite: Adjusted estimate (95% CI) | Sub-elite: Adjusted estimate (95% CI) | Group effect (95% CI) | p value |
| --- | --- | --- | --- | --- |
| RT (ms) | 388.7 (358.5, 419.0) | 389.0 (358.7, 419.3) | differences = −0.3 (−49.2, 48.7) | 0.992 |
| False response (%) | 3.9 (1.3, 11.2) | 3.4 (1.0, 10.5) | OR = 1.15 (0.21, 6.42) | 0.875 |
| Peak acceleration (m/s²) | 23.0 (19.7, 26.4) | 15.9 (12.5, 19.2) | differences = 7.2 (1.8, 12.6) | 0.009 |
| Peak jerk (m/s³) | 772.6 (639.9, 905.2) | 442.6 (310.0, 575.2) | differences = 329.9 (115.7, 544.2) | 0.003 |
| Premature reaction (%) | 6.0 (3.1, 11.5) | 18.9 (11.5, 29.5) | OR = 0.28 (0.10, 0.75) | 0.012 |
| Overall EMG RMS | 1.34 (1.10, 1.64) | 1.68 (1.37, 2.05) | Ratio = 0.80 (0.59, 1.09) | 0.151 |

Note. Values are age-adjusted estimates with 95% confidence intervals (CIs). For RT, peak acceleration, and peak jerk, group effects are adjusted mean differences (Elite − Sub-elite). For premature reactions and false responses, adjusted estimates are model-based probabilities expressed as percentages, and group effects are reported as odds ratios (ORs) for Elite versus Sub-elite. EMG RMS values were log-transformed for analysis; the group-specific estimates are back-transformed adjusted geometric means on the original normalized scale, and the group effect is reported as a geometric mean ratio (Elite/Sub-elite). Overall EMG RMS represents the model-based marginal estimate averaged equally across the flexor carpi radialis, biceps brachii, and anterior deltoid on the log scale and subsequently back-transformed. Models were additionally adjusted for opponent, attack, stimulus timing, and trial order. All age-adjusted mixed-effects models converged successfully. A boundary warning was issued for the RT model; however, the estimated random-effect variances remained positive and the group-effect estimate was stable relative to the corresponding unadjusted model. No convergence warnings were observed for the other age-adjusted models. CI, confidence interval; OR, odds ratio; RT, reaction time; EMG, electromyography; RMS, root mean square.
